# Financial frauds’ victim profiles in developing countries

**DOI:** 10.3389/fpsyg.2022.999053

**Published:** 2022-10-11

**Authors:** Eldad Bar Lev, Liviu-George Maha, Stefan-Catalin Topliceanu

**Affiliations:** ^1^Doctoral School of Economics and Business Administration, Alexandru Ioan Cuza University of Iasi, Iași, Romania; ^2^Department of Economics and International Relations, Faculty of Economics and Business Administration, Alexandru Ioan Cuza University of Iasi, Iași, Romania

**Keywords:** financial fraud, white-collar crime, victim’s behavior, investment decision, fraudulent scheme, developing countries

## Abstract

Recently, the variety of the financial frauds have increased, while the number of victims became difficult to estimate. The purpose of this paper is to present the main profiles of financial frauds’ victims using a reviewing method. The analysis captures the main theoretical and empirical background regarding the motives and circumstances of becoming a victim, the dynamics of several social and demographical characteristics of this type of victims, as well as a sample of relevant case studies from some developing countries. The main finding is that, in literature, most of the victims are male people of different ages, employed, married or single, regardless the level of education. For developing countries such as China, India and Nigeria, the majority of victims act out of naivety and desire to escape from poverty, while some victims from Latin America, China and Nigeria are influenced by greed and lack of empathy, without thinking of further consequences for their families and friends involved. Moreover, most of the victims are convinced to invest in financial schemes by family members, friends, or acquaintances.

## Introduction

In the last decades, economic crime has been thoroughly investigated at the international and national levels, with an emphasis on corruption and bribery. Economic or financial crime refers to illegal acts committed by an individual or a group in order to obtain an illegal material, economic, financial or professional gain. The economic crime attracts criminal organizations thanks to the low chance that these frauds would be discovered (Milosevic, 2016). On the other hand, individuals and organizations alike are not mindful of the risk of falling victim to economic crimes.

Taking into consideration only the territory of the European Union, the criminal activities have multiplied, over 80% being related to trade in drugs, organized property crime, investment, online and other frauds, trafficking in human beings and migrant smuggling (Europol, 2021). Fraud schemes refer to the intentional deception or intent of a person to deceive using techniques, instruments and false and deceptive pretexts through which fraudsters intend to determine victims to voluntary and unlawful transfer to them values, goods, money or iniquitous advantages. In exchange for these transfers, fraudsters promise economic, financial or material benefits for victims, which, in fact, do not exist or cannot to be granted. In this context, fraud schemes are one of the main serious and organized crime activities in the EU, while legal business structures are used in more than 80% of the criminal networks (Titus et al., 1995; Europol, 2021). There are various types of fraud schemes, including financial fraud, excise, mass marketing, benefit, payment-order, procurement, value-added tax, insurance, EU subsidy and loan and mortgage frauds. Financial fraud is based on social engineering techniques, meaning the attempt to obtain sensitive or personal information about victims that can be used for fraudulent purposes (Titus et al., 1995; Europol, 2017).

In the last years, the financial frauds have expanded and diversified, reflecting the growing creativity of the fraudsters and the permanent motivation of victims for rapid high returns. There are conceptual confusions between financial and investment frauds. Financial fraud includes a wider range of illegal acts such as phishing, identity theft or insurance scam, while investment fraud is a component of the financial category (Harvey et al., 2014). The most common investment frauds include boiler room, pyramid and Ponzi schemes. Also called “pressure room scams,” the boiler room schemes involve putting pressure on potential victims to invest in fictitious or low-value stocks. Fraudsters use cold calls for victims and present their business using fake references. On the other hand, pyramid and Ponzi schemes are similar, but in the case of pyramid, the first investors need to recruit new investors in order to generate profits (Europol, 2017). Despite the diversification of fraudulent programs, Ponzi schemes have retained their original method, to which fraudsters have come up with innovations. Charles Ponzi developed the original scheme in the 1920s, who promised a 50% return for those who decide to invest in international mail coupons. This fraud involves a promise of high returns with limited risks or none, while the fraudster uses the funds in personal or illegal purposes. The fraud operator pays the old investors using some of the funds received from new investors in order to lure more victims (Frankel, 2012). The operation of a Ponzi and pyramid schemes is based on a constant flow of new participants to the scheme. Therefore, the duration of the network is directly proportional to the number of victims, which means that the greater the number of victims, the longer the pyramid structure lasts (Titus et al., 1995).

The victimology remains one of the most studied fields when it comes to analyzing financial scams. When a scheme is uncovered, thousands of people who had thought that their money was invested in a safe and profitable business lose everything. Financial frauds are a type of business that has increased exponentially in all parts of the world, having in recent times a greater capacity for expansion. It is due to the difficult times in which people live, plagued by major economic crises, high unemployment rates and job insecurity. A large number of desperate people are looking to gain high rates on their investment in a short period. Such opportunities become very tempting for many. Other factors are the globalization of communications, and markets, the easy manipulation through the Internet, which means that a fraudulent company does not have to create a physical headquarters in a certain country.

The purpose of this study is to present the main profiles of financial frauds’ victims and the factors that support the tendency of victims to be attracted by these fraudulent schemes. The method used to conduct this paper implies a review of the primary sources (books and articles) in order to establish the main theoretical framework regarding the victimology of financial schemes and its determinants. Further, the method involves a descriptive and detailed analysis of the main profiles of financial frauds’ victims in developing countries by collecting, synthetizing and describing examples and case studies.

This study is structured as follows. Section Motives and circumstances for becoming victim presents the main motives and circumstances for becoming a victim of financial frauds, considering both contextual circumstances and psychological underpinnings. Further, the next three parts describe the theoretical and empirical background regarding the main components of the victim profile analysis, in terms of social-demographic characteristics, level of cooperation with the offenders, as well as other relevant items. Section Profiles of victims in developing countries provides relevant examples and case studies regarding the profiles of victims of financial frauds in developing countries. At the end, a discussion section is provided.

## Motives and circumstances for becoming victim

Researchers built models and surveys in an attempt to understand how different factors come together and influence the tendency to become victims of fraud attempts.

Lee and Soberon-Ferrer (1997) have suggested that cognitive deficiency and social interaction influence the tendency to become victims of frauds, both being related to biological, economic, sociological and psychological features. The cognitive deficiency refers to the limited ability of some individuals to process information, which make them more vulnerable to become victims. The cognitive ability is influenced by aging process, knowledge and experiences of individuals. On the other hand, social interaction refers to the quality of social networks and the level of social isolation. A low level of social interaction, whether is about a lifelong social isolation or a contextual one (due to a negative events), makes people more vulnerable to become victims. Moreover, the psychological isolation plays a higher role in this process than physical aloneness.

Lea et al. (2009) compared victims and non-victims regarding the degree of chance of falling victim because of poor judgment, and found that victims’ tendency to poor judgment is higher than among non-victims. From this, the authors concluded that victims are subject to persuasion in general and not necessarily to a specific type of fraud in which they were involved. They have identified various factors associated with poor judgment regarding financial fraud, and they divided them into two groups. First group includes motivational factors, such as:

- Motivation of basic human needs and desires (fear, greed, and visceral influence);
- Search for excitements in risk-taking;
- Lack of self-control;
- Low motivation for information processing;
- Reciprocity as the tendency to return a favor for favor;
- Commitment and consistency: fraudster take advantage of the need of a victim to make contact and engagement and then turning to him to invest money.

The second group refers to cognitive factors, such as:

- Positive illusions as the tendency of the individual to perceive himself in a favorable light and to appreciate his abilities;
- Prior knowledge in a specific field and overconfidence in the ability to make the right decisions;
- Low cognitive abilities (especially for elderly people);
- Social proof;
- Using norms of conduct such as reaching out to others and behaving politely;
- Authority: as the tendency to accept authority.

Contrary to the common perception that the behavior of the victims is irrational, Harvey et al. (2014) have interviewed 31 victims of investment fraud and found that they actually made rational decisions. The actions taken by victims of investment fraud are in fact rational, given the combination of the information accessible to them and circumstances of their lives at the time of the fraud. Victims testified an active attitude to ensure that they are subject of a real investment opportunity. They sought information and consulted with experts, family or friends, but the information they were able to gather was inconclusive, and even supported the legitimacy of what later turned out to be fraud. Victims testified that they decided to invest in financial schemes based on a combination of financial, family, and psychological circumstances at the time of the fraud:

1. Financial circumstances:
  - Financial resources available (even a change of financial situation);
  - Perceptions regarding systems and financial institutions;
  - The social and financial networks, including its quality.
2. Family circumstances:
  - The pressure to increase the family income fast;
  - The need for a long-term financial security for family.
3. Psychological circumstances

Dove (2018) proposes a model that presents various personal and cognitive elements that influence the tendency to fall victim to frauds. First, there will be preconditions for fraud, or personal circumstances in the life of the potential victim, for example, lack of time to review the offer or any personal need that the offer may meet. Victims are influenced by the fact that frauds motivate the victim’s basic needs and desires by promising to earn a slightly higher profit from the effort he has invested. Second, the potential victim will perceived an offer to invest in fraudulent schemes as attractive when it sounds credible or limited in some way (discount for 1 day only). Once the potential victim is under the impression that the offer is credible, that person may cooperate immediately due to certain cognitive factors, such as being impulsive or easily persuaded. Moreover, frauds gain authority by fraudsters’ claim that a bank or other authoritative figures back the offer. These aspects impair the victims’ judgment, leading to a higher tendency of invest in fraudulent schemes. On the other hand, some victims may be skeptical or reflective. They will decide to test the offer even though it sounds credible or to think about it before investing. Moreover, victims may be aware that their past impulsive decisions led to unfavorable outcomes. With such experiences, they will delay decision making, which may reduce emotional involvement and will gain extra time for a more careful examination.

Considering the models developed by literature, this paper proposes an approach to the motives and circumstances of becoming victim of financial frauds in accordance with Harvey et al. (2014) and Dove (2018). Moreover, this paper suggests that the financial and family circumstances to be treated in common as contextual circumstances, while the cognitive and personal elements to be presented as psychological underpinnings.

### Contextual circumstances

When victims decide to invest their money, they considered both financial and non-financial aspects, in order to assess whether it was a rational decision with regard to their life circumstances. The financial considerations focused mainly on the ratio between the risk in the investment and the potential profit. Some of the victims interviewed by Harvey et al. (2014) were under the impression that the risk was low compared to the high potential profit. Even if some victims were aware of the high level of risk, they were willing to risk in a small amount of their investment savings. There were those who preferred an investment that yielded high profits to an investment in a banking program that yielded lower profits. On the other hand, the non-financial considerations focused on the non-financial benefits that victims would gain from their investment, for instance, refinement of financial skills and greater control over their investment than investing through a bank. Some victims reported that “enjoy the risk,” being a pattern of behavior conceived by Lyng (2005) as “edgework” and described as the temptation to take risk consciously when the incentive is the experience itself. Indeed, victims mentioned the risk, among other things, as exciting, fun and gambling.

The financial circumstances include the available financial resources of the victims (even a change in their financial situation), the perceptions regarding systems and financial institutions and the social and financial networks. Everyone had initial money to invest, but some victims interviewed by Harvey et al. (2014) are getting into debt later in order to continue the investment or to repay the fund. Some of the victims reported that their financial situation was static when the crook-initiated contact with them. Moreover, there are victims who experienced a change in personal financial circumstances shortly before the fraudster’s contact, whether it was a benefit (increase in financial resources) or a deterioration (decrease in financial resources). This change was a significant factor that explains why these individuals fell victims. Those who experienced a sudden increase in financial resources were debating where to invest their money in order to multiply profits. On the other hand, some victims experienced a sudden reduction in liquid funds and were pressured to quickly find additional financial resources. Some victims stated that the advice received from financial and social networks did not help them in the investment decision, but rather contributed to invest in fraudulent schemes. Regarding the perceptions of financial systems and institutions there are two types of victims (Harvey et al., 2014). The optimistic ones believed that there was significant regulations with a clear enforcement. They decided to invest in fraudulent schemes assuming that their money is safe as long as they transfer it to a bank account or because the fraudsters provided an account number managed by old and well-established banks. On the other hand, the pessimistic victims were suspicious of banks and in general of the global financial system. They considered that fraudsters are more trustworthy, while the government interventions led to the collapse of the business, which was in fact a fraudulent scheme. They assumed that if the government not enforced the closure orders, the financial scheme would have continued to yield them high profits. Moreover, very religious people tend to question the legitimacy of any source of authority other than members of their religion, including the authority of the government (Frankel, 2012).

The family circumstances refer to the pressure of increasing the income immediately for the benefit of the family economy and to the responsibility for providing financial security to the family in the long-term. Lifestyle factors such as an active social life and working tend to expose people to a higher risk of frauds (Muscat et al., 2002). Victims interviewed by Harvey et al. (2014) described situations in which they felt immense financial pressure or responsibility:

- They became the primary or sole breadwinner of their children;
- They wanted to work fewer hours so that they would have more time to spend with their children;
- They had to stop working to care for an elderly parent;
- They had to fund a large expense, for example, a wedding;
- They were constrained to assist their children to achieve financial security;
- They had to assist the extended family members with a more precarious financial situation.

Victims felt that they did not have enough time to think about before the investment decision due to the pressure exerted on them to reach a decision quickly.

### Psychological underpinnings

The main psychological factors that support the tendency to be victim of frauds are related to gullibility, risk tolerance, the level of self-control, the level of prior knowledge in financial field, the character traits and the ability to discern between true and false information.

Starting with gullibility, this can be considered a form of stupidity, defined by the situation when “someone engages in dangerous social or physical activity even though there are warning signs or he has questions about them that have not been addressed and should have worried him” (Greenspan, 2009, p. 22). Foolish people tend to believe even when something is too good to be true. Gullibility means trusting based on insufficient evidence, and acting on emotion, hope, or desire. Foolish people are more dependent, and therefore weaker than skeptical and suspicious people who trust themselves more than others. The crooks act as if they are invincible, radiating power and control over what is happening. In the face of these messages, the victims feel weak, insecure, and full of doubts. They consider that are incompetent, less smart and not sophisticated enough. Moreover, when victims fail to transcend these feelings of inferiority, they prefer to hide and disguise them, adopting values and behaviors similar to those of the crooks (Frankel, 2012). In this context, some ethical issues can be addressed, if the victim understands the warning signs or even more is aware that it is the case of a fraudulent scheme, not being emotionally affected by the fact that the desired potential financial gain implies losses for more other victims.

Another cognitive factor is risk tolerance. The combination of gullibility and tolerance of risk creates a powerful tendency to take risks and become a victim of frauds. People who have a high tolerance of risk take risks in general and not necessarily in the financial field, choosing, for example, mountain climbing as a hobby. Van Wyk and Benson (1997) found that people with a positive approach to financial risk are at higher risk of being a target by crooks. Schoepfer and Piquero (2009) suggested that people with risky behaviors are more likely to become victims of fraud. Lea et al. (2009) believed that some fraud victims identify the risks involved in investing but take the risk nonetheless, hoping it would pay off for them. If the guaranteed profit is large enough, the risk will be perceived as worthwhile. Wood et al. (2018) suggested that higher benefits are associated with higher willingness to invest in fraudulent schemes, while high risks discourage individuals for such attempts. The importance of the risk tolerance in this context is emphasized by Harvey et al. (2014), who presented four types of victims of investment frauds according to this characteristic. The risk averse investors are the first type, being the most skeptical and reserved individuals, with low risk appetite and less than 1 month being engage in an investment scheme. They record a low emotional impact and no financial losses, have experience with investments and connected financial social networks. In contrast, adventurers have a positive thinking and a high-risk appetite, having the longest time of engagement in an investment scheme and a medium-high financial and emotional impact. Even if they have financial social networks, may not use it. The third type is represented by the dabblers, having a medium-high risk appetite. Usually, their engagement time is less than 6 months, while the financial and emotional impact is low medium. Providers, who record the biggest financial and emotional impact, even if they are individuals with a low-medium risk appetite, represent the last group.

Low self-control is another determinant of attractiveness for financial frauds. People with low self-control tend to take risks due to the urge for immediate gratification and are more vulnerable to falling as victims (Langenderfer and Shimp, 2001; Schreck et al., 2006; Holtfreter et al., 2008). In addition, Holtfreter et al. (2010) discovered a positive association between low levels of self-control and propensity to fall victim to fraud, while for Modic and Lea (2012) both low self-control and impulsivity play an important role in fraud compliance. Impulsivity influences the likelihood of falling victim to fraud by impairing the decision-making process (Bayard et al., 2011). Pratt et al. (2014) suggested that those with a low level of impulsivity took less risk and were less willing to gamble, while self-control is a consistent predictor of the likelihood of falling victim in general. Schreck (1999) indicated that that low self-control leads to lack of premeditation and perseverance. Lack of premeditation leads to errors in decision-making, while victims with low levels of premeditation have a poor ability to plan and to predict future consequences of their actions. This suggests that those victims are more willing to share their personal information because they do not think the consequences (Modic and Lea, 2012).

The level of prior knowledge in financial field plays an important role in attracting fraud victims, even if the opinions are divided regarding this role. AARP (1999), Langenderfer and Shimp (2001), and Kadoya et al. (2021) found that the absence of prior knowledge about frauds or in a field related to a particular fraud (e.g., financial or investing), increases the chance of falling victim to the scam. Almost 75% of the interviewed victims by AARP (2007) have a low level of financial investing knowledge. In contrast, Lea et al. (2009) assert that prior knowledge is what increases the chance of falling victim, because in the face of such knowledge the victim behaves with less caution. Many times, victims of investment frauds have prior knowledge in the field of financial investment (Xiao and Porto, 2021; Yang et al., 2022). Rebovich and Layne (2000) found that victims of investment frauds are more financially literate than the general population.

The ability to discern between true and false information influences the attractiveness of victims for financial frauds. Here there are two types of persons, those with a low “need for understanding” and those with a high level. People with a higher “need for understanding” were more persuaded by a message addressed to cognition, compared to those with a low “need for understanding,” who were more persuaded by an impressive message. Those with a high level tend to process information through greater cognitive effort. Cacioppo et al. (1986) found that persons with a low “need for understanding” were less likely to differentiate between weak and strong messages, were less affected by the quality of arguments, and invested less effort in examining evidence than participants with a high “need for understanding.” On the other hand, Kaufman et al. (1999) said that those with a low “need for understanding” examined evidence more deeply when they believed the source was unreliable, while people with a high “need for understanding” were less affected by the source’s credibility. Unreliable sources increase motivation among those with a low “need for understanding” to invest more effort in information processing. Nevertheless, because fraud offenders often impersonate trusted sources, those with a low “need for understanding” are more likely to become victims of the frauds than they are likely to process the information relevant to the offer.

Information processing is also affected by personal traits. The message is perceived as more convincing when it is adjusted to individual’s personality (Haddock et al., 2008). Some character traits allow for deception and other not. Modic and Lea (2012) found that six personality traits explain the fraud compliance, such as premeditation, extraversion, openness, sensation seeking, urgency and self-control. Harvey et al. (2014) discovered a list of factors regarding the character traits that enable the success of the fraud such as decisiveness, extroversion, low self-esteem, positivity, honesty, the tendency to trust too much and the tendency to gamble. Some character traits indicate openness to opportunities and willingness to take high risks, such as adventure, propensity for addiction, and aspiration to succeed. The tendency to believe too much in people allows deception, while some victims felt themselves under psychological pressure when the crook contacted them. They were emotionally vulnerable to fall victim to the fraud, especially due to a clinical depression or the loss of someone close. Their mental state impaired their decision-making process during the referral from the crook or during the fraud. On contrary, some victims reported that they became victims precisely when they were happy and peaceful. Other participants enumerated character traits that made them unable to interrupt the conversation with the crooks, such as the tendency to help others and a polite attitude toward others. Certain behaviors have the potential to increase the tendency to fall victim to a fraud, such as joining online groups, making online purchases, and making donations (Titus and Gover, 2001). In contrast, some character traits such as discretion, curiosity, thrift, skepticism and seriousness serve as a shield and inhibit fraud attempts. The survey participants conducted by Harvey et al. (2014) compare their approach to life to the one of those around them, noting that others are more careful with their money or do not strive for financial success and therefore their chances of falling victim are low. Victims who did not transfer money to crooks described the personal traits that they thought stopped the fraud attempts such as caution, skepticism and discretion. Some victims were careful and reflective and decided to delay the offer of investing in fraudulent programs in order to gain more time for examination (Dove, 2018).

The tendency of fall victim to frauds can be expressed through a model of “Big Five personality factors,” which include five major personality characteristics such as openness to change, conscientiousness, extraversion, agreeableness and neuroticism (Tupes and Christal, 1992; Parrish et al., 2009; Modic and Lea, 2012). The openness to change means that a person is openness to experiences, while his conscientiousness will reduce the chance of falling victim because a person with this characteristic tends to obey safety warnings if he has received them. At the same time, an extrovert is more likely to share information with others, while agreeableness involves trust. Finally, the neuroticism will reduce the chance of falling victim because a neurotic person will refuse to share personal information on a web platform and will even navigate less online sites (Parrish et al., 2009). Modic and Lea (2012) indicated that both openness and extraversion have an important influence in fraud compliance.

## The dynamics of the socio-demographic characteristics of victims

Socio-demographic dynamics of the financial frauds’ victims include the main characteristics in terms of age, gender, education, marital and professional statuses. Some scholars found that only age and education play an important influence in predicting the tendency of falling victims in personal frauds (Titus et al., 1995; Van Wyk and Benson, 1997; Kerley and Copes, 2002). Lee and Soberon-Ferrer (1997) have suggested that age, education and marital status have a significant influence on predicting victims, while age has the largest effect. However, some scholars have determined profiles of victims considering the main demographic characteristics mentioned above (Shadel and Schweitzer-Pak, 2007; Zunzunegui et al., 2017).

The literature is mixed regarding whether younger or elderly people are more vulnerable of being victims of financial frauds (Table 1). Studies that examined the victims’ profile until the 2010s have shown that both young and old can be victims of frauds. However, most of the studies focused on the study of victims since 2013 revealed that most of victims are old people. Titus et al. (1995) suggested that young people are more likely to become victims due to their lower income and a higher level of receptiveness to opportunities for rapid income growth, while older people have a higher tendency to report frauds and, for this reason, the fraudsters avoid them. In addition, the risk of adults falling victim to a fraud is three times lower than the risk for young people. Van Wyk and Benson (1997) declared that young people are more willing to be victims of financial frauds because scammers believe that young people tend to take more risks. Schoepfer and Piquero (2009) highlighted the tendency of young people to take greater financial risks, further increases their propensity to become victims. Young people tend to fall victim to proposals for business opportunities and work from home, mysticism, and network frauds. On the other hand, the elderly population tends to fall victim to the frauds regarding high-risk investments and providers of services that come at home (Button et al., 2009). Deliema et al. (2020) indicated that the odds of being victim of investment fraud grow with 4% with each year that age increases. Rebovich and Layne (2000) have interviewed 1,1,69 people and 60% of them believed that older people are the most likely to become victims of frauds.

**Table 1:** Profiles of victims according to age across time.

Author	Period studied	Most of victims
Lokanan (2014)	1984–2008	Old people
Van Wyk and Benson (1997)	1989–1994	Young people
Titus et al. (1995)	1990–1991	Young people
Lee and Soberon-Ferrer (1997)	1993	Old people
Shichor et al. (1996)	1994	Old people
Kerley and Copes (2002)	1994	Young people
AARP (1999)	1998	Old people
Schoepfer and Piquero (2009)	1999	Young people
Muscat et al. (2002)	2000	Young people
Pascoe et al. (2006)	2005	Young people
AARP (2007)	2006	Old people
Bolimos and Choo (2017)	2008–2013	Old people
Experian (2010)	2009	Young people
Harvey et al. (2014)	2013	Old people
Button et al. (2014)	2014	Old people
Xu et al. (2022)	2015	Old people
Zunzunegui et al. (2017)	2015–2016	Old people
Deliema et al. (2020)	2016	Old people
Kadoya et al. (2021)	2020	Old people
Yang et al. (2022)	2022	Old people

In terms of gender, men tend to fall victim to the foreign making frauds, network frauds, high-risk investments and land investments, while women are vulnerable on network scams, health and weight loss products that promise miracles, mysticism scams, and false career advancement offers (Button et al., 2009). However, almost all scholars found that most of the victims of financial frauds are male (Table 2). Lee and Soberon-Ferrer (1997) found that older women are more vulnerable of becoming victim than older man, but the situation is reversed for younger groups.

**Table 2:** Profiles of victims according to gender across time.

Author	Period studied	Most of victims
Lokanan (2014)	1984–2008	Male
Lee and Soberon-Ferrer (1997)	1993	Both male and female
Shichor et al. (1996)	1994	Male
Pascoe et al. (2006)	2005	Male
AARP (2007)	2006	Male
Shadel and Schweitzer-Pak (2007)	2006–2007	Male
Experian (2010)	2009	Male
Bolimos and Choo (2017)	2008–2013	Male
Harvey et al. (2014)	2013	Male
Button et al. (2014)	2014	Male
Xu et al. (2022)	2015	Male
Zunzunegui et al. (2017)	2015–2016	Male
Deliema et al. (2020)	2016	Male
Wood et al. (2018)	2018	Male
European Commission (2020)	2019	Male
Kadoya et al. (2021)	2020	Male
Yang et al. (2022)	2022	Male

Generally, education is seen as a factor that influences the tendency of becoming victim because individuals use the skills gained through formal schooling in decision-making, even for financial decisions (Lee and Soberon-Ferrer, 1997). Burke et al. (2022) indicated that the people’s wiliness to become victims of investment frauds could be reduced through education, especially the online educational interventions. However, the literature is mixed regarding the level of education in profiling victims and no clear pattern was established across time (Table 3). On the one hand, high-educated persons are more likely to become victims of frauds for several reasons even if they know how to assess risks better than less educated people do (Titus et al., 1995; Van Wyk and Benson, 1997). One of the reasons is their own perception that they are educated and experts in their field and they apply this judgment to the fields in which they are not very prepared. They believe that they are protected from fraud, because of their intelligence. Kerley and Copes (2002) and Schoepfer and Piquero (2009) extend the analysis, arguing that education is a variable highly associated with crime reporting and suggesting that those with higher education levels are more likely to report fraud to authorities. Copes et al. (2001) argue that the decision of reporting a fraud is influenced by factors such as level of education, marital status, age and whether the offender was a stranger to the victim. On the other hand, some scholars indicated that people with low level of education have a higher risk to become victims of financial frauds. Lee and Soberon-Ferrer (1997) found that the level of vulnerability decreases as the education and income level increase.

**Table 3:** Profiles of victims according to education across time.

Author	Period studied	Most of victims
Van Wyk and Benson (1997)	1989–1994	High educated
Titus et al. (1995)	1990–1991	High educated
Lee and Soberon-Ferrer (1997)	1993	Less educated
Shichor et al. (1996)	1994	High educated
Kerley and Copes (2002)	1994	Less educated
AARP (1999)	1998	Less educated
Shadel and Schweitzer-Pak (2007)	2006–2007	High educated
Zunzunegui et al. (2017)	2015–2016	Less educated
Wood et al. (2018)	2018	Less educated
European Commission (2020)	2019	High educated
Yang et al. (2022)	2022	High educated

According to marital status, it seems that the literature is also divided, regardless the period studied (Table 4). Some scholars argue that married people are more vulnerable to fall victim, considering that first victims of someone affected by a pyramid fraud will be the victim’s family and friends, which are unaware of the deception. The most intimate circle is usually the most prone to the extension of the base pyramidal. Generally, most of the victims are small savers, looking for an alternative to investing their money, relying on advice from family and friends (Shadel and Schweitzer-Pak, 2007). On the other hand, single people are more vulnerable of being victims than married ones considering the social isolation and feelings of loneliness (Kadoya et al., 2021).

**Table 4:** Profiles of victims according to marital status across time.

Author	Period studied	Most of victims
Lee and Soberon-Ferrer (1997)	1993	Single
AARP (2007)	2006	Married
Shadel and Schweitzer-Pak (2007)	2006–2007	Married
Zunzunegui et al. (2017)	2015–2016	Married
Deliema et al. (2020)	2016	Married
Wood et al. (2018)	2018	Single
Kadoya et al. (2021)	2020	Single

There are fewer studies regarding the professional status than other demographic features (Table 5). However, most of studies indicated that most of victims are employed. Shadel and Schweitzer-Pak (2007) developed two surveys for two different years having contrary findings. In the first survey, most of victims were retired and unemployed, while the second one was in accordance with most of scholars.

**Table 5:** Profiles of victims according to professional status across time.

Author	Period studied	Most of victims
Lokanan (2014)	1984–2008	Employed
AARP (2007)	2006	Employed
Shadel and Schweitzer-Pak (2007)	2006	Retired and unemployed
	2007	Employed
Button et al. (2014)	2014	Employed
Zunzunegui et al. (2017)	2015–2016	Employed

## The level of cooperation

Researchers have investigated if there is cooperation or facilitation from the victim in the frauds and have tried to create a profile of the degree of cooperation. It is necessary to emphasize that this discussion is looking to draws attention to the role that victims can have in assisting fraudsters and developing frauds, not to invoke moral judgments about victim blame. In this perspective, there are ethical reasons for questioning the status of victims for those investors with a high degree of cooperation with the offenders. Regarding this issue, the opinions are divided.

On the one hand, there are scholars who believe that victims are complicit in the fraud and their irresponsible attitude determines a full collaboration of victims with criminals in the development of frauds (Delord-Raynal, 1983; Titus and Gover, 2001; Button et al., 2009). Delord-Raynal (1983) believes that victims act out of greed and see fraudsters as accomplices who will help them to achieve gains. She created a link between the cooperation of victims with fraudsters and the tendency of reporting frauds. While victims avoid to report frauds because of shame over being deceived, another reason can be the fear of expose the victim’s dishonest intentions. Titus and Gover (2001) define victims as “careless.” For example, among victims of identity theft it was found that some threw bank reports in the trash, uploaded personal information to social networks and did not secure their personal computer. Others have even been warned by the bank that they are about to make a transfer to an unreliable source, but have chosen to ignore the warning (Button et al., 2009).

On the other hand, given that financial crimes are committed using persuasion tactics, rather than force, many victims feel guilty about being fallen victim and are even perceived as such by others. Authorities often perceive victims as sharing the blame with the perpetrators (Shichor et al., 2001; Levi, 2008). Van Wyk and Benson (1997) place some of the responsibility on the victim claiming that a decent person will not fall prey to a fraudster. On contrary, Harvey et al. (2014) have argued that victims are not responsible for frauds committed against them.

However, it is not possible to generalize these studies, because the types of fraud are varied and so are the types of victims. Thus, some scholars argue that there are different degrees of involvement by victims. Titus and Gover (2001) present three levels of involvement: significant cooperation, partial cooperation, and lack of cooperation. Victims in the non-cooperation group are defined as having the lowest degree of involvement. An example of a victim belonging to this group is a company manager who is unaware that his personal details have been stolen and that false loan applications have been filed on his behalf. Victims in a partial collaboration group are defined as having a higher degree of involvement than the previous group. They cooperate with the crook, but passively. For example, victims will provide personal details in phishing messages or be persuaded to buy worthless shares following a random phone call. Victims in a significant collaboration group are defined as having the highest degree of involvement. To some extent, they even show active involvement. For example, victims who respond to marketing ads or who are actively seeking to invest in programs with a high potential for fraud such as job opportunities from home that require money to be invested in advance. Titus (1999) detailed these groups by defining types of collaboration. There are victims who initiate contact with the offender (responds to marketing ads, visiting websites, etc.). Some victims use to share personal information with the offender, while others allow the offender to turn a business relationship into a personal one. Another type of victims share personal financial information with the offender, while some victims allow the offender to create a version of events for the purpose of fraud.

Cialdini (2001) presents principles of influence that may cause even the most intelligent people to cooperate with fraudsters, as follows:

- Reciprocity as the desire to return a favor;
- Commitment and consistency as a tendency to honor obligations even when the original motive for granting the obligation no longer exists;
- Social proof view as a desire to imitate those we trust;
- Authority as the tendency to obey authoritative figures, even if they are conducted in an unacceptable way;
- Affinity as the acceptance to be persuaded when the persuader is a popular figure.

Affinity crime is defined as a crime in which there is a certain affinity between victims and fraudsters on ethnic, professional or religious grounds, or a common social circle. The prevailing opinion is that a person will not deceive people with whom he has an affinity, and therefore the offender exploits the trust created toward him (Springer, 2020).

## Other relevant characteristics in victim profiling

### The degree of awareness

Profiles according to the degree of awareness of fraud consider four levels of awareness (Button et al., 2009):

- victims who are not even aware being scammed;
- victims who are aware of being scammed, but choose not to report it to the authorities;
- victims who are aware of this and report to authorities;
- victims who find it difficult to believe that it was indeed a scam.

Many people are unaware that they were victims of a fraud until they discover it following a request from the authorities. This is valid for certain types of fraud, for example, in illegal lottery games that are very likely to win anyway, and in charitable organizations that rely on the victim’s tendency to donate without making sure that the request came from a legal organization (Fraud Advisory Panel, 2006).

However, in most cases the victims will find out that it is a fraud. Some of them will report to the authorities and some will avoid it. Almost 59% of the victims interviewed by Rebovich and Layne (2000) have chosen not to report falling as victim, while 41% of victims from EU have decided to reported fraud to no one (European Commission, 2020). Kerley and Copes (2002) have suggested than only 10% of victims report frauds to police, while the number increases at 22% for victims involved in multiple fraud attempts. Scholars found that the tendency of no reporting frauds has various explanations. Button et al. (2009) mentioned confusion, ambiguity of fraud and embarrassment as factors of no reporting. Some victims are confused as to where they should report frauds; others believed that they were victims of an unfortunate investment rather than fraud. Mason and Benson (1996) continued the list of factors and specified that the low reporting rate by victims is linked to perceptions of responsibility, level of loss, social networks and justice process. Victims avoid reporting frauds if they feel embarrassed, blame themselves for falling victims or tend to believe of sharing responsibility in part or in full with the fraudsters. Some victims avoid reporting because want to hide the losses incurred from their social networks (family and friends). On the other hand, the social network’s attitudes toward the fraud may encouraged victims to report frauds or not. In addition, victims avoid reporting when they incurred small losses or when they believed that the criminal justice process is untrustworthy (Titus, 1999). Copes et al. (2001) and European Commission (2020) suggested that the tendency of victims for reporting frauds increases as the losses incurred grow. Regarding the criminal justice process, Reisig and Holtfreter (2007) highlighted than less than 50% of American victims trust that the authorities will successfully solve the fraud cases in which they are involved. Bolimos and Choo (2017) indicated that victims between 2008 and 2013 reported more than 57% of online frauds. Copes et al. (2001) found that the victims’ tendency to involve the law in pursuing frauds is linked to morphology and cultural context as derived factors from “The Black’s theory of law.” According to this, morphology suggests that strangers are more willing to use the law than natives, while people that are more educated are more likely to involve law in fraud detection.

The fourth group consists of victims who do not believe that this is indeed a fraud. These are chronic victims because they respond to repeated requests by the fraudsters (Button et al., 2009). They believe in the legality of the company, invest their money and try to induce others to follow them with the illusion that the investment is beneficial for all the victims involved (Spalek, 2016).

### Losses incurred and frequency of falling victim

Other profiles can be determined according to the extent of the losses incurred and the number of times they fell victim to a fraud, grouping them in chronic victims, large-scale, low-volume and unidentified victims. The number of chronic victims is low, and mass marketing often harms them. They lose large parts of their income or savings usually in recurring losses of relatively low sums of money (Shichor et al., 2001). On the hand, the number of the large-scale victims is high, and they usually fall victim once or a few times and lose large sums of money. Some of them will report the fraud and some will avoid it. The highest number of all victims are those low-volume and unidentified victims, of which some are aware of fallen victim to a fraud, while those who have lost very small sums of money are unaware (Button et al., 2009).

In the survey conducted by Titus et al. (1995), 58% of respondents declared that were victim one or more times during their life. Moreover, during the past 12 months, 31% of them have declared falling victim one or multiple times. Pascoe et al. (2006) found that more than 75% of victims had experienced multiple fraud attempts.

As regarding the losses incurred, there were victims who did not suffer financial losses despite being subjected to fraud (Titus et al., 1995; Harvey et al., 2014). However, most of victims reported losses in various currencies (Table 6). In the survey conducted by Zunzunegui et al. (2017) the average losses incurred by victims were 60,660 euros. For victims interviewed by Kerley and Copes (2002) the average amount lost is almost $270 and exceeds $750 for repeat victims. Bolimos and Choo (2017) indicated that the average amount lost was $4,000.

**Table 6:** Losses incurred by victims across time.

Author	Period studied	Losses incurred by most victims
Lokanan (2014)	1984–2008	<$25,000
Titus et al. (1995)	1990–1991	$26–$1,000
Shichor et al. (1996)	1994	<$30,000
Shadel and Schweitzer-Pak (2007)	2006	>$1,000
AARP (2007)	2006	$100,000–$500,000
Experian (2010)	2009	£59,000
Harvey et al. (2014)	2013	£50,000
Button et al. (2014)	2014	£1,000–£10,000
Zunzunegui et al. (2017)	2015–2016	€300,000
European Commission (2020)	2019	€1–€500

## Profiles of victims in developing countries

Victims in developing countries are presented considering their contextual circumstances and their main profiles. Several countries were selected from literature, such as Bolivia, China, Columbia, India, Malaysia, and Nigeria.

### Contextual circumstances

The main circumstances that lead to the increase of financial fraud’s victims are related to economic hardship. Many victims are married and, considering both financial and family circumstances, they need money to support their families in the short term (Dreber et al., 2009; Apicella et al., 2014). Indian victims are looking for extra money to improve their standard of living or to build their financial base to improve their economic conditions. In addition, the economic conditions and poverty encourage the existence of financial frauds in Nigeria as victims are looking to improve their standard of living (Jack and Ibekwe, 2018). In contrast, lack of empathy for other investors and greed are factors that support the tendency of being attracted by financial frauds in China, Nigeria and Latin America.

Financial frauds have begun to thrive in China in recent decades, that the authorities have defined the phenomenon as a real threat to social order. One of the reasons for the blossoming of the frauds is loose regulation on financial entities operating in the network, alongside greed and a desire to get rich that have become a major driving force among Chinese society (Dor, 2017). Regardless of the poor economic conditions identified for Nigeria, Obamuyi et al. (2018) have associated greed with one of the main motivations for the rise of financial schemes. The expectation of earning high returns in a short period was one of the motivating factors for participating in financial schemes along with the low and deteriorating living standards in Nigeria. When many participants received high short-term returns, others were fascinated by the return and joined the fraud. Existing and new investors focused on the returns and did not question how the high returns were realized. At the same time, most of the victims in countries from Latin America, such as Bolivia or Columbia, are low-income and small savers, who are looking for an alternative investment for their savings other than the ones offered by local banks (Heinemann and Verner, 2006). In many cases, they do not pay attention to the qualification of who is going to manage their money, nor whether the businesses or investments in which they would be participating is legally registered business, as long as what was promised is fulfilled. Often, the income obtained by the victims from the fraudster is “reinvested” in the same scheme, since the trust in the organizer increases once the latter pays what had been agreed upon. However, some investors remain committed to recruiting new participants, for which they receive a commission or some rather benefits (Monroe et al., 2010). Since what matters to them is making money, they also have no misgivings in recruiting other people in order to benefit from the commissions, without exposing the risks to the new investors.

These two opposing situations raise a number of ethical issues, while, unfortunately, no ethical sensibility is found in the literature. Some victims are acting with naivety and gullibility out of the pure desire to advance from a very poor condition, hoping that their decisions will bring extra money in short time. Other victims are acting out of lack of empathy and greed. Even if some investors or victims know about the financial fraud in which they are involved, they choose not to address the warning lights as long as they received their pay. Some people invest in fraudulent schemes even though they suspect it was a scam or knew it for sure, when they still hope to profit from the investments of others, by recruiting additional investors and knowing that their money will flow to current investors. Thanks to their greed and to their desire to maintain professional and economic status, some investors may falsify data and take illegal actions for fear of losing their status and wealth. The highest level of lack of empathy is reflected when they are trying to recruit family, friends or acquaintances to participate in the fraud (Frankel, 2012). They imitate the behavior of fraudsters, being able to endanger the safety of those around him, even the family, out of the desire to increase their wealth. They act ignoring the ethical consequences arising from their behavior.

Regarding the financial social networks, most of victims in developing countries decided to invest in financial schemes after being convinced by family, one acquaintance or friend that they trusted. For countries from Latin America, victims usually rely on the advice of relatives or close friends (Heinemann and Verner, 2006). In Malaysia, some investors have become victims through close ties to members of an exclusive group. Victims, like retirees, are often members of local religious groups, local neighborhoods, and other hobby or personal interest groups. These victims trusted the fraudster because of his good behavior and the long relationship between them (Piaw et al., 2019). For Nigeria, the participation of victims in investment schemes includes trusting the recommendation of a friend and charities provided by the fraudsters. Moreover, some churches encouraged their members to invest in financial programs that were later found to be fraudulent (Aluko and Olawuni, 2021).

One of the main psychological factors that supports the tendency of becoming a financial fraud victim in developing countries is the level of financial knowledge. One of the reasons behind the popularity of financial frauds among the people of India is the low level of financial awareness and financial literacy within population. Moreover, over half of agricultural households in India are economically excluded from formal financial institutions. The same situation is valid for developing countries from Latin America. Most financial scheme operators seem to take advantage of the loophole and to offer a simple investment process, promising a high and easy return to people in agricultural communities, who have no access to banking facilities (Heinemann and Verner, 2006). Two types of victims can be identified in Malaysia in accordance with financial education. Many victims in Malaysia lack financial awareness and are easily defrauded. They have no basic understanding of financing and choose to invest in financial schemes out of unfamiliarity and naivety. However, there are also victims who have some experience and knowledge in financial matters and have joined the financial schemes out of greed (Piaw et al., 2019). The lack of a sound legal and regulatory environment contribute to the operation of financial schemes in African economies, this case being also valid for Nigeria (Jack and Ibekwe, 2018). For China, individuals are an easy prey for frauds because the Chinese markets opened in the 90s and therefore population has little experience in money management, familiarity with financial risks and the ability to choose financial products (Dor, 2017). In addition, an increased level of financial literacy acts as a defending factor against becoming a victim of financial fraud. Despite the financial literacy, the risk orientation is emphasized in predicting financial fraud victims for China, which are more likely to classify themselves as optimistic with a high tendency of risk-taking. This trend is particularly pronounced among those who have fallen into a fraud more than once (Cheng, 2016).

### Socio-demographic features for developing countries

From an age perspective, victims in developing countries are either young or old. For China, the results are mixed. Most of the Indian and Nigerian victims are young middle age, considering that the use of the internet attracted many young victims, especially those interested in cryptocurrencies. On the other hand, most of Malaysian victims are those who have expected or have recently had a windfall, such as older people. Windfall wins typically include large sums of money given to recently retired employees through a mandatory savings plan known as the Employee Provident Fund. New retirees are attracted to participating in financial schemes for two reasons. First, the expected returns would serve as a source of lucrative income, and second, retirees would feel that putting a small portion of the windfall into an investment plan is not considered risky since they still have plenty of cash left over (Piaw et al., 2019). In Bolivia, the potential victims for financial schemes are retirees with low income and a large marginal capacity to save. The victims invest their capital in different pyramid companies and they start from a small amount of saving (Heinemann and Verner, 2006).

There are two common versions in China of financial scams; one in which investors are recruited through the promise of a quick profit without risk provided they recruit additional investors and the other in which young people are taken captive and forced to recruit investors. This situation among the young people is causing a stir on social media in China due to a number of suicides that have occurred within its framework (Dor, 2017). On the other hand, people in their 60s in China are therefore more likely to be victims of financial frauds considering that many Chinese think of boosting their retirement income by investing in financial schemes. The average age is close to the retirement age of most Chinese people, suggesting that victims on the verge of retiring invested more money in financial schemes and tended to invest more times than younger people. People on the verge of retiring or retirees tend to feel economically pressured as they anticipate expensive medical outlays and increased living expenses. The frustration of people in this age group translates into a strong predisposition to find lucrative investments in the short term (Cheng, 2016).

In gender terms, most of Indian victims were married men considered that the male members of the family make financial decisions. In contrast, most of the Chinese victims of financial schemes were females, because in many Chinese families, women take care of managing the family’s financial affairs. However, the total amount invested by the women in financial schemes was almost equal to the total amount invested by the men. The women tended though to invest multiple times in small amounts, while men were more liable to invest a large amount at once. This finding implies that men appear to be more amenable to risk taking than women, who took small steps in their investment decision (Cheng, 2016).

In terms of education and professional status, most of the victims in India are usually employed and have a formal education (academic degree or higher). Educated people are more prone to fall as financial fraud victims considering that recently frauds in India were based on cryptocurrencies. Therefore, only those who read about cryptocurrencies tried to invest in them. In Bolivia, victims are generally small savers who were looking for investment alternatives for their small of savings. Unemployed workers with minimum wages are potential victims (Heinemann and Verner, 2006). In contrast, for China, people with higher socioeconomic status tend to be more prone to be deceived (Cheng, 2016).

## Discussion

Financial fraud has increased around the world, having in recent times a greater capacity for expansion due to economic insecurity and lack of financial knowledge. Victims are attracted by financial frauds considering their contextual circumstances (financial and family) and their psychological underpinnings. While the financial circumstances refer to available financial resources, financial situation and social networks, the family ones consider the pressure to secure the family’s financial future. On the other hand, the tendency of being victim of frauds is related to gullibility, risk tolerance, the level of self-control, the level of prior financial education, the degree of discernment and the character traits.

The victims’ profiles of financial frauds can be determined in different terms.

From a socio-demographic perspective, the most used features in profiling victims are age, gender, education, marital and professional statuses. The literature is mixed regarding age, education and marital status. Scholars found that both younger and elderly people, neither married nor single, with high or low education are vulnerable of being victims of financial frauds. However, in the last years, studied revealed a tendency of considering the most of victims of frauds as old people. In terms of gender and professional status, most of scholars agreed that most of victims are male and employed. Therefore, male people, regardless the age, employed, married or single, with high or low education can form a complete profile of victim from demographic point of view.

In terms of level of cooperation from the victim in the frauds, there are scholars who believe that victims share blame in part or in full with the fraudsters. However, we believe that deception is more a fault of the fraudsters, while victims were involved in apparently profitable processes that later turned out to be fraudulent. Even if there are people who became victims because of their lack of attention or responsibility toward their money or personal and family data, these victims cannot be blamed of cooperating with fraudsters. In the worst case, it can be added that their irresponsible actions facilitated the fraudulent process, while the fraudsters took advantage of the victim’s weakness, but this does not make them as guilty as the fraudsters. Therefore, from our perspective, there is no doubt that the fraudsters are responsible for choosing their targets and for manipulating the victims.

In terms of degree of awareness, profile of victims varies from people who are unaware of being victims to individuals who do not believe in being scammed. However, victims have various motives for choosing whether to report frauds, some of them being related to the frequency of falling victim and losses incurred.

As regarding the victims of financial frauds from developing countries, there are different profiles. In India, most of victims are young people, especially married men, looking for extra returns in order to improve their living conditions. The same purpose is met in China, but the main types of victims are females or people close to the retirement age. A similar profile in terms of people retired is valid for Malaysia and Bolivia, where older people are more vulnerable to be victims of financial frauds. Most of Asian victims have a low level of financial education, being attracted in financial schemes due to unfamiliarity, naivety and desire to escape from poverty. Despite the desire of improving their living conditions, some victims from China, Latin America and Nigeria are attracted in financial frauds by greed and lack of empathy, without thinking of further financial, emotional and ethical consequences of their unfair behavior.

Another finding of this paper is that most of the victims decided to invest after being convinced by family, acquaintances or friends that they trusted. In some countries, there is a herd behavior as a tendency of victims to follow others in their circle. In Malaysia and Nigeria, some of the victims are attracted through the affinity in terms of religion, ethnicity or personal interest.

The number of victims can be decrease by growing government regulations and by increasing population information and prevention actions, especially among low-income people. Moreover, countries must find tools to improve the living conditions for their citizens and to reduce the economic and social negative effects in times of economic uncertainty so that victims no longer be interested in fraudulent ways of winning. Future research may imply a fraud victimization survey for a representative sample of specific cases from developing countries.

## Author contributions

All authors listed have made a substantial, direct, and intellectual contribution to the work and approved it for publication.

## Funding

Authors are thankful to Romanian Ministry of Research, Innovation and Digitization, within Program 1—Development of the national RD system, Subprogram 1.2—Institutional Performance—RDI excellence funding projects, contract no. 11PFE/30.12.2021, for financial support.

## Conflict of interest

The authors declare that the research was conducted in the absence of any commercial or financial relationships that could be construed as a potential conflict of interest.

## Publisher’s note

All claims expressed in this article are solely those of the authors and do not necessarily represent those of their affiliated organizations, or those of the publisher, the editors and the reviewers. Any product that may be evaluated in this article, or claim that may be made by its manufacturer, is not guaranteed or endorsed by the publisher.
